# Glucagon-like peptide-1 receptor agonists to modulate neuroinflammation and neurogenesis

**DOI:** 10.4103/NRR.NRR-D-25-01144

**Published:** 2025-12-30

**Authors:** Rosalie Elvira, Eng King Tan, Zhi Dong Zhou

**Affiliations:** National Neuroscience Institute of Singapore, Singapore; Signature Research Program in Neuroscience and Behavioral Disorders, Duke-NUS Medical School, Singapore; Department of Neurology, Singapore General Hospital, Singapore

Glucagon-like peptide-1 receptor agonists (GLP-1RAs), originally developed as drugs for type 2 diabetes and obesity, exhibit therapeutic effects for various neurological disorders recently, with multiple neuroprotective mechanisms. The GLP-1RAs therapy has been applied to acute ischemic stroke, where they limit neuroinflammation, stabilize the neurovascular unit, and reduce infarct volume. The GLP-1RAs drugs have also been tested for Wolfram syndrome, where they mitigate endoplasmic reticulum (ER) stress and inhibit neuronal apoptosis. Preclinical analyses demonstrate that GLP-1RAs can modulate neuroinflammation processes, including reprogramming of microglia towards anti-inflammatory phenotypes, suppression of nucleotide-binding domain, leucine-rich–containing family, pyrin domain–containing-3 (NLRP3) inflammasome activation, suppressing pathological astrogliosis, and rebalance of peripheral immunity. Furthermore, GLP-1RAs were identified to enhance synaptic plasticity, improve cognitive deficits, and promote hippocampal neurogenesis via regulation of brain-derived neurotrophic factor/cAMP response element-binding protein (BDNF/CREB) and insulin-like growth factor 1 (IGF-1) pathways. However, embedded multimodal biomarkers (e.g., TREM2, neuroimaging, and neurogenesis markers) are required for further clinical translation and clinical trial investigation of target engagement, dosage optimization, and patient stratification. Future prioritizing mechanism-focused studies in the niche will help unlock the potential of GLP-1RAs as disease-modifying therapies for human neurological disorders.

Glucagon-like peptide-1 (GLP-1) is an incretin hormone released postprandially to regulate glucose metabolism. It enhances insulin secretion in a glucose-dependent manner, suppresses glucagon release, slows gastric emptying, and promotes satiety. These actions are mediated via the GLP-1 receptor (GLP-1R), which is comprehensively expressed in the pancreas, gastrointestinal tract, brain, and other tissues. GLP-1RAs are synthetic analogs developed to harness benefits of GLP-1 in treating type 2 diabetes and obesity. These agents provide metabolic and cardioprotective benefits.

**Figure 1:**
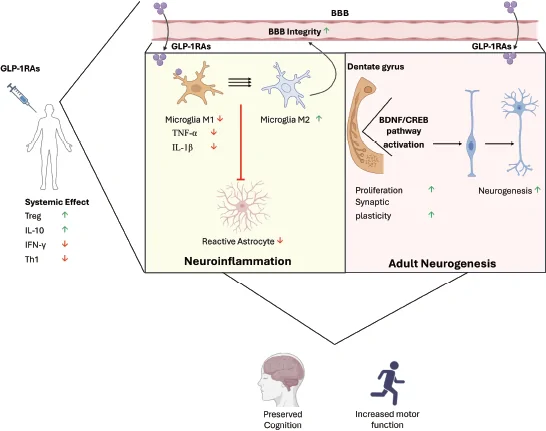
Mechanistic actions of GLP1 receptor agonists (GLP1RAs) in neuroinflammation and neurogenesis. Systemically administered GLP1RAs (e.g., liraglutide and exendin4) cross the blood–brain barrier (BBB) and exert multiple neuroprotective effects. In the neuroinflammatory axis (left), GLP1R activation promotes a shift in microglia from proinflammatory M1 to antiinflammatory M2 phenotypes, downregulates proinflammatory cytokines (TNF-α and IL-1β), and suppresses reactive astrocyte activity—collectively enhancing BBB integrity. In the neurogenic axis (right), GLP1RAs increase expression of BDNF and activation of the CREB pathway, enhancing synaptic plasticity and promoting adult neurogenesis in the dentate gyrus. These mechanisms converge to preserve cognitive and motor functions in models of neurodegenerative disease. Created with BioRender.com. BBB: Blood–brain barrier; BDNF: brain-derived neurotrophic factor; CREB: cAMP response element binding protein; GLP-1RA: glucagon-like peptide-1 receptor agonist; IL-1β: interleukin-1 beta; IL-10: interleukin-10; IFN-γ: interferon-gamma; M1 microglia: classically activated, pro-inflammatory microglia phenotype; M2 microglia: alternatively activated, anti-inflammatory microglia phenotype; mtTFA: mitochondrial transcription factor A; PGC-1α: peroxisome proliferator activated receptor gamma coactivator-1 alpha; SIRT1: sirtuin-1; Th1: T helper type 1 cell; TLR-1: Toll-like receptor 1; TNF-α: tumor necrosis factor alpha; Treg: regulatory T cell.

Beyond their metabolic effects, growing evidence points to GLP-1RAs as promising drug candidates for neurodegenerative diseases, owing to their neuroprotective, anti-inflammatory, and pro-regenerative properties. Ongoing trials in Alzheimer’s disease (AD) and Parkinson’s disease (PD) are assessing clinical outcomes (Zhou et al., 2025), yet key mechanisms such as neuroimmune modulation and enhanced neurogenesis have so far been explored mainly in preclinical models. While AD and PD are the ultimate targets, we contend that the path to demonstrating disease modification for GLP-1RAs lies not in large, heterogeneous trials in these complex diseases, but in strategically prioritizing niche indications with high mechanistic fidelity. We propose acute ischemic stroke and Wolfram syndrome as exemplar contexts where a defined onset, clear pathophysiology, and measurable short-term outcomes offer a powerful platform to accelerate proof-of-concept testing. Validating target engagement and refining biomarker frameworks in these high-signal settings is a critical first step before broader application. Through the lens of these indications, this perspective will focus on two underexplored yet pivotal mechanisms: neuroimmune modulation and adult neurogenesis (**Figure 1**), and outline a biomarker-driven roadmap for future translation.

**Expanding indications — ischemic stroke & Wolfram syndrome:** We propose that acute ischemic stroke and Wolfram syndrome represent ideal, albeit underexplored, clinical test beds for GLP-1RAs, precisely because their pathologies align directly with the drugs’ core mechanisms of action. Their defined etiologies and acute or monogenic nature offer a more tractable setting for proving mechanistic efficacy than the complexity of sporadic AD or PD.

GLP-1RAs offer multifaceted neuroprotection in ischemic stroke through both central and systemic mechanisms. By activating GLP-1R in cells of the central nervous system (CNS), they reduce infarct volume, suppress oxidative stress/neuroinflammation, and preserve blood–brain barrier (BBB) integrity. These actions involve key signaling pathways such as PI3K/Akt, cAMP/PKA/CREB, and nuclear factor kappa-light-chain-enhancer of activated B cells (NF-κB) inhibition, which collectively limit apoptosis and promote neurogenesis (Vergès et al., 2022). Peripherally, GLP-1RAs improve glycemic control, lower blood pressure, enhance lipid metabolism, and stabilize atherosclerotic plaques, reducing stroke risk factors. By integrating metabolic correction with direct neurovascular modulation, GLP-1RAs emerge as promising therapeutic candidates in ischemic stroke prevention and recovery.

Wolfram syndrome (WS), a rare autosomal recessive disorder driven by *Wfs1* gene mutations, uniquely combines neuronal ER stress with pancreatic β-cell failure. In an aged rat model of WS, the GLP-1RAs, such as liraglutide, demonstrated significant neuroprotective effects by targeting key pathological mechanisms of neurodegeneration. Liraglutide treatment over 6 months reduced neuroinflammation and ameliorated ER stress in the inferior olive—a brainstem region critically affected in WS. This was evidenced by decreased expression of ER stress markers such as GRP78 and reduced neuronal swelling, suggesting improved cellular homeostasis (Seppa et al., 2019).

Importantly, liraglutide preserved retinal ganglion cells and prevented optic nerve axon degeneration, which are hallmarks of WS-related visual decline. Stereological analyses revealed increased neuronal counts in the dorsal nucleus of the inferior olive in treated rats, indicating protection against age-related neuronal loss. These effects are likely mediated through GLP-1R–dependent signaling pathways that enhance neuronal survival, reduce oxidative stress, and suppress glial activation. The study also noted delayed progression of hyperglycemia, reinforcing dual benefit of liraglutide in managing both metabolic and neurodegenerative aspects of WS (Seppa et al., 2019). Translation requires validation in human models/cohorts due to species differences and absence of clinical trials.

By mitigating ER stress, reducing neuroinflammation, and preserving neuronal integrity, liraglutide offers a promising therapeutic strategy for slowing neurodegeneration in Wolfram syndrome.

**Glucagon-like peptide-1 receptor agonists and neuroinflammation:** Beyond metabolic effects, we posit that the reprogramming of neuroimmune responses is a central pillar of GLP-1RA-mediated neuroprotection, a mechanism that must be explicitly targeted and measured in future trials.

Although originally developed for peripheral metabolic control, several GLP-1RAs can cross BBB at therapeutic doses, allowing them to directly engage with CNS targets, and modulating neuroinflammation. Neuroinflammation is a complex inflammatory response within the brain or spinal cord, and is increasingly recognized as a critical factor in the progression and pathology of various neurological disorders, including neurodegenerative diseases such as AD and PD. Indeed, chronic microglial activation and astrocytosis are now understood to drive amyloid-β accumulation, tau phosphorylation, α-synuclein aggregation, and dopaminergic neuron loss in AD and PD models, making immunomodulation a key therapeutic target.

Once within the CNS, GLP-1RA exerts direct effects on resident immune cells, particularly microglia. GLP-1RAs activate microglial GLP-1 receptors, driving a shift from a pro-inflammatory (M1) status to an anti-inflammatory (M2) phenotype. This reprogramming alleviates neuroinflammation and associated behavioural deficits. For instance, in diet-induced obesity mice, exenatide treatment down-regulates scavenger receptor A4 and suppresses NF-κB, which effectively reduces the generation of proinflammatory mediators and cytokines such as tumor necrosis factor alpha (TNF-α) and interleukin (IL)-6. GLP-1RA also suppressed nucleotide-binding domain, leucine-rich–containing family, pyrin domain–containing-3 (NLRP3)-mediated pathways, resulting in reduced anxiety and improved locomotor activity. In addition, during sepsis-induced encephalopathy, GLP-1RAs attenuate ER stress, further suppressing inflammatory cascades and apoptosis in microglia.

Furthermore, GLP-1RA, such as exendin-4 and semaglutide, can engage GLP-1Rs following the peripheral injection on synaptically connected neuronal populations rather than on microglia or circulating immune cells, thereby avoiding direct modulation of Toll-like receptor signaling in innate immune effectors. Activation of these neuronal GLP-1Rs triggers a downstream neuroendocrine cascade via α1-adrenergic and δ-/κ-opioid receptor circuits, which suppresses systemic and cerebral release of pro-inflammatory cytokines in response to diverse Toll-like receptor agonists (Wong et al., 2024).

Beyond modulating resident immune cells, GLP-1RAs also play a critical role in fortifying the BBB and mitigating astrocyte activation through effects on the neurovascular unit. GLP-1RAs coordinate endothelial cells, pericytes, and astrocytes to preserve tight junction architecture and barrier selectivity, while stimulating production of vascular endothelial growth factor and other trophic factors that drive angiogenesis and boost cerebral perfusion. Under diabetic or oxidative-stress conditions, they counteract pathological nitration of the GLP-1 receptor, restoring receptor signaling in pericytes and stabilizing pericyte–endothelial contacts. At the same time, GLP-1RAs inhibit matrix metalloproteinases that degrade the basement membrane and maintain expression of tight junction proteins such as occludin and claudin-5, reducing paracellular leak (Chen et al., 2024). The resulting improvement in vascular function limits peripheral immune cell infiltration and subsequent astrocyte activation, thereby creating a resilient, neuroprotective milieu.

Through these combined actions: reprogramming microglia, modulating astrocytes and the neurovascular unit, and rebalancing peripheral immunity, GLP-1RAs interrupt the sustained neuroinflammatory state that drives neuronal loss and synaptic dysfunction. Importantly, in preclinical models of AD and PD, GLP1RAs not only reduce hallmark proteinopathies (amyloid-β plaques, neurofibrillary tangles, and α-synuclein inclusions) but also preserve cognitive performance, motor coordination, and dopaminergic neuron survival. These findings have propelled several GLP1RA candidates into earlyphase clinical trials for neurodegenerative disorders.

**Glucagon-like peptide-1 receptor agonists and neurogenesis:** We argue that the pro-neurogenic effects of GLP-1RAs are not merely ancillary but are synergistic with their anti-inflammatory actions, together creating a self-reinforcing cycle of CNS resilience that must be leveraged therapeutically.

Preclinical studies demonstrate that GLP-1RAs (e.g., exendin-4 and liraglutide) directly stimulate neural progenitor proliferation and synaptic remodeling via BDNF/CREB and IGF-1 pathways, promoting neural progenitor proliferation, dendritic complexity, and synaptic plasticity in mice dentate gyrus. For instance, exendin 4 restores long term potentiation and reverses obesity induced memory deficits by rescuing BDNF/CREB signaling in rodent models. A recent systematic report summarizes consistent up-regulation of neurogenesis markers (doublecortin^+^ cells) and synaptic proteins (synapsin 1) across diverse disease models (Au et al., 2025).

Indirectly, a major mechanism of GLP-1RA in neurogenesis involves the regulation of mitochondrial function and oxidative stress. GLP-1RAs optimize mitochondrial resilience—a critical determinant of neuronal survival. They enhance mitochondrial biogenesis via the peroxisome proliferator-activated receptor gamma coactivator 1-alpha/nuclear respiratory factor 1/mitochondrial transcription factor A axis and combat oxidative stress by activating the GLP-1R/cAMP/PKA and nuclear factor erythroid 2-related factor 2/heme oxygenase 1 pathways. This reduces mitochondrial-derived reactive oxygen species in AD hippocampus, while improving respiratory function through SIRT1 activation and ER-mitochondria crosstalk. These mechanisms collectively preserve energy metabolism and genomic integrity in neural progenitors.

Functionally, enhanced neurogenesis and mitochondrial health translate to improved spatial memory and reduced anxiety-like behavior in AD, aging, and metabolic syndrome models. Critically, GLP-1RAs enable this recovery by augmenting cerebrovascular perfusion and blood-hippocampus barrier integrity, ensuring delivery of neurotrophic factors (IGF-1), metabolic substrates (glucose and lactate), and oxygen to neurogenic niches (Au et al., 2025). This vascular-metabolic support fosters neuronal maturation and circuit integration.

Taken together, GLP-1RAs deliver a synergistic neurogenic and neuroprotective strategy in neurodegeneration: by bolstering synaptic plasticity, restoring mitochondrial health, and preserving vascular and metabolic homeostasis, they underlie the improved memory, learning, and motor outcomes seen in preclinical AD and PD models. Ongoing clinical trials will determine whether these multimodal effects can translate into disease modification and improved quality of life for patients.

Critically, GLP-1RAs orchestrate synergistic crosstalk between neuroinflammatory and neurogenic pathways. Suppression of microglial activation and pro-inflammatory cytokines (e.g., IL-1β and TNF-α) removes inhibitory signals that impair neural progenitor proliferation and synaptic plasticity (Au et al., 2025). Conversely, enhanced neurogenesis reinforces anti-inflammatory feedback loops via BDNF-mediated microglial quiescence and astrocyte modulation. This bidirectional interplay — where immunomodulation enables neuroregeneration, and newborn neurons sustain inflammatory homeostasis — creates a self-reinforcing cycle of CNS resilience that disrupts core neurodegenerative cascades.

**Limitations and future directions:** Although GLP-1RAs demonstrate consistent anti-neuroinflammatory and pro-neurogenic actions in multiple preclinical models, most mechanistic data remain rodent-centric. Species differences in BBB permeability, immune cell composition, and GLP-1 receptor expression complicate direct extrapolation to humans, and validated, standardized markers of adult human neurogenesis are still lacking (Qi et al., 2023). Accordingly, early proof-of-mechanism studies in humans are essential to determine whether preclinical pathways translate into clinically meaningful biology.

The clinical literature to date highlights a substantive translational gap. Though early-phase trials report improved cognitive/motor scores, such as slow motor deterioration and improved cognitive function in PD by lixisenatide (Meissner Wassilios et al., 2024) and AD by liraglutide (Edison et al., 2021), several Phase II/III programs, including trials of exenatide in AD and PD, failed to meet primary endpoints, despite evidence of biological activity in some studies. These discrepancies can be plausibly attributed to inadequate CNS target engagement at doses repurposed from metabolic indications, heterogeneous patient selection and late-stage enrollment, outcome measures that are insensitive to early biological change, and the absence of embedded pharmacodynamic biomarkers demonstrating brain target modulation. Indeed, recent clinical data show that a GLP-1RA can modulate plasma inflammatory proteins in AD patients, indicating target engagement without necessarily producing clinical benefit under the tested conditions (Koychev et al., 2024).

To operationalize such a strategy, we recommend a prioritized, multimodal biomarker panel for early human studies that triangulates target engagement across fluid, imaging, and functional domains. Fluid measures should include cerebrospinal fluid and plasma markers of neuroimmune engagement (sTREM2 and YKL-40), neuroaxonal injury (NfL), and disease-relevant pathology where applicable (amyloid beta 42/40 and p-tau), together with multiplex cytokine panels (IL-6, TNF-α, and IL-1β) and neurotrophic factors (BDNF and IGF-1). Peripheral immune profiling (flow cytometry for monocyte subsets, Treg/Th1 balance, and functional innate assays) provides complementary, readily accessible readouts. Neuroimaging should prioritize modalities that reflect immune or neurogenic biology, translocator protein-positron emission tomography for microglial activation (used with careful genotyping and interpretation given translocator protein polymorphism and limited cellular specificity), disease-specific positron emission tomography ligands when available, and magnetic resonance imaging surrogates such as hippocampal cerebral blood volume/volumetry or magnetic resonance spectroscopy as pragmatic proxies for neurogenic or metabolic change doublecortin, while a robust neurogenesis marker in rodents, has limited reproducibility and validation in adult humans. Short, sensitive cognitive and motor batteries (including remote/digital assessments) should be paired with biomarker endpoints to correlate biological change with function. Crucially, no single marker is definitive; early trials should therefore combine modalities, pre-specify analytic pipelines, and harmonize assays across sites to ensure reproducibility and regulatory acceptability.

Operational and design considerations are equally important. Dedicated pharmacokinetic/cerebrospinal fluid bridging studies must establish CNS penetration and inform dose selection because repurposed metabolic doses may not achieve sufficient brain exposure; alternative delivery strategies (for example, intranasal administration) and head-to-head dose escalation experiments should be considered to optimize CNS engagement. Adaptive trial designs with predefined “go/no-go” criteria — such as a prespecified percentage change in a primary biomarker (e.g., ≥ 20%–30% change in sTREM2 or a meaningful reduction in NfL) combined with demonstrable cerebrospinal fluid drug detection and acceptable safety — would enable rapid decision-making and limit costly late-phase failures. We suggest short biomarker readouts (4–12 weeks) be required to justify progression to larger clinical efficacy studies.

Finally, both preclinical and clinical results are context-dependent: semaglutide and tirzepatide show limited effects in some AD models, and the mixed clinical outcomes of exenatide highlight the influence of genetic background, disease stage, co-morbid metabolic states, and dosing/exposure. These mixed data support a niche, mechanism-first approach, prioritizing indications and patient subsets with high mechanistic fidelity (for example, genetically defined disorders or acute settings where BBB disruption facilitates CNS access), alongside robust biomarker validation and CNS exposure confirmation. By embedding multimodal biomarkers, confirming CNS pharmacokinetics, and using adaptive, mechanism-driven designs, the field can more reliably assess whether the promising preclinical neuroimmune and neurogenic mechanisms of GLP-1RAs translate into durable human benefit.

**Conclusion:** GLP-1 receptor agonists represent a paradigm-shifting therapeutic class with multiple neuroprotective mechanisms, including maladaptive neuroinflammation through microglial reprogramming, astrocyte modulation, and peripheral immune rebalancing, while simultaneously enhancing neuroregeneration via BDNF-driven hippocampal plasticity and activation of neural progenitors. While rodent models provide compelling evidence of these benefits, clinical translation remains bottlenecked by inadequate proof of target engagement in humans. To unlock their disease-modifying potential, we urge prioritization of mechanism-focused trials that leverage multimodal biomarkers in short-term studies, enabling clear readouts of pharmacodynamic effects.

Equally important is the stratification of patients by neuroinflammatory or neurogenic endophenotypes, which will help identify those most likely to respond and avoid diluting trial outcomes with heterogeneous cohorts. In conclusion, we advocate for a strategic pivot in the clinical development of GLP-1RAs for neurological disorders. Rather than pursuing large, costly trials in heterogeneous common neurodegenerative diseases upfront, the field should first prioritize targeted, mechanism-focused studies in niche indications such as acute ischemic stroke and Wolfram syndrome. These settings provide a clear path to: (1) definitively confirm human target engagement using the multimodal biomarkers we have outlined; (2) establish CNS-optimized dosing regimens; and (3) generate early proof-of-concept efficacy signals in a tractable timeframe. Success in these focused efforts will de-risk subsequent investment in AD and PD trials and provide the validated biomarker tools necessary to identify responsive patient subpopulations within those complex diseases. This disciplined, stepwise approach is essential to unlock the true disease-modifying potential of this promising drug class.

*We thank NNRC and Duke-NUS Medical School for funding supports of this work.*

*This work was supported by the Open Fund Large Collaborative Grant (MOH-OFLCG24may-0004) and the Singapore Translational Research (STaR) Investigator Award (NMRC/STaR/0030/2018) (to EKT); the Open Fund-Individual Research Grant (OF-IRG, MOH-001506), Clinician Scientist-Individual Research Grants (CS-IRG) (MOH-001091, CIRG23jul-0006), HLCA2024 (HLCA24Mar-0019), and SingHealth-Duke-NUS Academic Medicine Position Grant (to ZDZ).*

**Additional file:** *Open peer review report 1.*

OPEN PEER REVIEW REPORT 1
