# Supplementary material for: Glucagon-like peptide-1 receptor agonists to modulate neuroinflammation and neurogenesis
Source: Neural Regen Res. 2025 Dec 30;21(9):4229–30. doi: 10.4103/NRR.NRR-D-25-01144 (PMC13557675; doi:10.4103/NRR.NRR-D-25-01144)
Supplement: Supplementary file 1 [file NRR-21-4229_Suppl1.pdf]

OPEN PEER REVIEW REPORT 1

Name of journal: Neural Regeneration Research
Manuscript NO: NRR-D-25-01144
Title: Glucagon-like peptide-1 receptor agonists to modulate neuroinflammation and neurogenesis
Reviewer's Name: Ivan Koychev
Reviewer's country:UK

COMMENTS TO AUTHORS

This perspectives article explores the neuroprotective potential of GLP-1 receptor agonists (GLP-1RAs), with emphasis on two underexplored mechanisms: neuroimmune modulation and adult hippocampal neurogenesis. The manuscript concisely synthesizes evidence from preclinical and early clinical studies, highlighting applications in ischemic stroke, Wolfram syndrome, and neurodegenerative disorders (e.g., AD, PD). The authors also stress the need for biomarker-driven, mechanism-focused clinical trials to establish target engagement and translational relevance.

Major Strengths

GLP-1RAs are a rapidly evolving therapeutic class, and positioning them within the neuroregeneration/neuroprotection field is highly valuable.

The manuscript avoids broad generalities and centers on two mechanistic pillars (neuroinflammation and neurogenesis), which provides clarity.

Integration of Preclinical and Early Clinical Evidence - Examples from ischemic stroke, Wolfram syndrome, AD, and PD models create a compelling rationale for translational exploration.

Figure 1 (page 11-12) - The schematic effectively illustrates how GLP-1RAs act across systemic, neuroimmune, and neurogenic axes to support cognition and motor outcomes.

Balanced Discussion - The limitations section appropriately cautions about rodent-centric data, species differences, and inconsistent trial outcomes.

Major Concerns

Overreliance on Preclinical Data - While acknowledged, the manuscript could further contextualize the translational gap. For example, exenatide's Phase II/III failures (AD/PD) merit more critical examination regarding why promising mechanisms fail clinically. Also, a clinical study that demonstrated effects of exenatide on inflammatory pathways associated with AD was not mentioned (doi: 10.1186/s13195-024-01573-x)

Biomarker Strategy Needs More Detail - The authors advocate multimodal biomarkers (CSF, imaging, immune profiling) but remain general. Specific candidate markers and their current validation status (e.g., TSPO-PET limitations, reproducibility of doublecortin as a neurogenesis marker) would strengthen the argument.

Scope of Indications - While ischemic stroke and Wolfram syndrome are discussed, the rationale for prioritizing these rare or acute contexts over common neurodegenerative diseases could be elaborated.

Terminological Consistency - Some sections alternate between "GLP1-RA," "GLP-1RA," and "GLP-1 receptor agonists." Standardizing terminology would improve readability.

Perspective vs. Review Balance - The manuscript sometimes reads like a mini-review rather than a forward-looking perspective. The editors may prefer clearer emphasis on future directions and conceptual frameworks rather than literature summary.

Minor Comments

page 2: Sentence structure is dense; consider breaking into shorter statements to enhance readability for non-specialist readers.

References: Several citations are very recent (2023-2025), which is excellent. However, a few seminal earlier works (e.g., Hölscher's pioneering GLP-1 neuroprotection studies pre-2015) could be acknowledged for completeness.
